# Thalamopeduncular Tumors in Pediatric Age: Advanced Preoperative Imaging to Define Safe Surgical Planning: A Multicentric Experience

**DOI:** 10.3390/jcm12175521

**Published:** 2023-08-25

**Authors:** Alberto D’Amico, Giulia Melinda Furlanis, Valentina Baro, Luca Sartori, Andrea Landi, Domenico d’Avella, Francesco Sala, Luca Denaro

**Affiliations:** 1 Academic Neurosurgery, Department of Neurosciences, University of Padova, 35122 Padova, Italy; 2 Section of Neurosurgery, Department of Neurological and Movement Sciences, University of Verona, 37100 Verona, Italy

**Keywords:** thalamopeduncular tumor, pilocytic astrocytoma, transcranial magnetic stimulation, thalamopeduncular syndrome, DTI MRI, brain mapping

## Abstract

**Background**: Thalamopeduncular tumors are challenging lesions arising at the junction between the thalamus and the cerebral peduncle. They represent 1–5% of pediatric brain tumors, are mainly pilocytic astrocytoma and occur within the first two decades of life. To date, the optimal treatment remains unclear. **Methods**: We retrospectively reviewed pediatric patients who underwent surgery for thalamopeduncular tumors in the Academic Pediatric Neurosurgery Unit of Padova and Verona from 2005 to 2022. We collected information on age, sex, symptoms, preoperative and postoperative neuroradiological studies, histological specimens, surgical approaches, and follow-up. **Results**: We identified eight patients with a mean age of 9 years. All lesions were pilocytic astrocytoma. The main symptoms were spastic hemiparesis, cranial nerve palsy, headache, and ataxia. The corticospinal tract was studied in all patients using diffusion-tensor imaging brain MRI and in two patients using navigated transcranial magnetic stimulation. The transsylvian approach was the most frequently used. A gross total resection was achieved in two patients, a subtotal resection in five and a partial resection in one. In three patients, a second treatment was performed due to the regrowth of the tumor, performing an additional surgery in two cases and a second-look surgery followed by adjuvant therapy in one. After the surgery, four patients maintained stability in their postoperative neurological exam, two patients improved, and two worsened but in one of them, an improvement during recovery occurred. At the last follow-up available, three patients were disease-free, four had a stable tumor residual, and only one patient died from the progression of the disease. **Conclusions**: Advanced preoperative tools allow one to define a safe surgical strategy. Due to the indolent behavior of thalamopeduncular tumors, surgery should be encouraged.

## 1. Introduction

Thalamopeduncular tumors are a new subgroup of lesions recently described by Puget et al. [1] that originate from the interface between the thalamus and the cerebral peduncles. They develop below a normal thalamus, pushing it upwards and displacing the corticospinal tract. Pediatric thalamopeduncular tumors represent less than 5% of all pediatric brain tumors. They can occur at all ages, rarely arise in adulthood, and mainly affect children in the first two decades of their life, with no gender preference. In the past, they were classified as tout court within a large group of tumors, defined as “thalamic”, “brainstem” or “basal ganglia” tumors, without considering their different features in terms of clinical or radiological presentation and therapeutic strategies. 

In most cases, the thalamopeduncular tumors are pilocytic astrocytoma. In the pediatric population, low-grade gliomas (LGG) have a very high chance of a long overall survival, reaching adulthood, and in the case of pilocytic astrocytoma and a histology without aggressive or infiltrative histopathological features, a complete recovery can be achieved with total resection, usually without the need for adjuvant oncological treatment.

If a more infiltrative tumor pattern is found, the recent molecular knowledge about genetic mutations such as KIAA1549-BRAF fusion and BRAF-V600E mutation could offer additional targets for therapies [2,3].

Frequently, thalamopeduncular tumors are responsible for a peculiar clinical syndrome named the “Thalamopeduncular syndrome of childhood”, characterized by progressive spastic hemiparesis associated with pyramidal signs. Hydrocephalus may be present due to the proximity of the lesion to the ventricular system. Other signs such as visual impairment, cranial nerve palsy, and focal seizures are less common [4]. 

Before the nineties, these tumors were considered inoperable, due to the deep and complex areas involved. The lack of adequate neuroimaging and intraoperative tools made the cost–benefit ratio of thalamopeduncular surgery unfavorable. In most cases, patients were referred for radiotherapy with a poor prognosis due to early relapse, malignant transformation, or cognitive impairment.

Nowadays, new advanced neuroimaging techniques such as diffusion-tensor imaging tractography (DTI MRI) and neuronavigated transcranial magnetic stimulation (nTMS), together with neuronavigation system and intraoperative neurophysiological monitoring, allow one to plan an accurate surgical strategy to obtain good control of the disease with low morbidity and a favorable long-term outcome [5,6,7].

## 2. Materials and Methods

We retrospectively reviewed pediatric patients who underwent surgery for thalamopeduncular tumors at the Academic Pediatric Neurosurgery Department of Padova and at the Academic Neurosurgery Department of Verona from 2005 to 2022. 

We collected eight cases, including four boys and four girls (M/F ratio 1:1) aged from 3 to 15 years with a mean age of 9 years. The clinical presentation at admission was progressive spastic hemiparesis in six patients, and in three of them, VII cranial nerve palsy was also observed. Other symptoms were ataxia in one case and headache in another one. In three patients, mild hydrocephalus was present (Table 1). For each patient, we assessed, sex, age at onset, the histopathological report (with Ki67 proliferation index), preoperative and postoperative brain MRI with gadolinium, DTI MRI to reconstruct the corticospinal spinal tracts, cortical motor mapping, white fiber reconstruction with nTMS (Nexstim^®^, Madison, WI, USA), the use of the neuronavigation system (Medtronic Stealth Station Navigation S7^®^, Lafayette, CO, USA), and the use of intraoperative neurophysiological monitoring (IONM).

The level of tumor resection was classified as a partial resection (<90% with the presence of residual tumor on postoperative MRI), a subtotal resection (>90% with small residual tumor on postoperative MRI), or a gross total resection (the absence of residual tumor on postoperative MRI).

Finally, we reported the surgical approaches performed, the additional surgery or eventual adjuvant therapies performed, the postoperative neurological status and the last clinical and neuroradiological follow-up available (Table 2).

## 3. Surgical Plan

Every patient underwent brain MRI with gadolinium (Gd) and DTI MRI tractography to reconstruct the cortical spinal bundles, and in recent cases, nTMS was used to reconstruct the cortical motor mapping.

Due to the deep sites and complex areas invaded by thalamopeduncular tumors, DTI MRI tractography (DTI) is an important tool used to identify the corticospinal tracts (CSTs) with the aim of planning a surgical approach and trying to obtain a gross total resection with respect to neurological function [8].

Neuronavigated transcranial magnetic stimulation (nTMS) was a recent neuroimaging tool introduced by the Academic Neurosurgery of Padova and Verona. nTMS is an innovative technique that allows one to obtain a preoperative functional mapping of the motor cortex, adding functional information on the CSTs with respect to the anatomic data obtained with DTI-MRI. nTMS requires collaboration from the patients, and for this reason, it is usually performed on adults [9].

**Figure 1:**
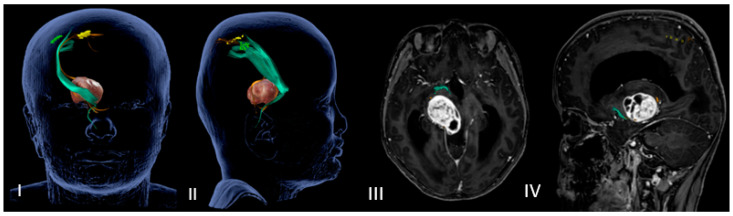
(**I**–**IV**) Neuronavigated transcranial magnetic stimulation pre-op study. (**I**,**II**) A 3D reconstruction of the nTMS data that shows cortical maps of the right spinal tract (CST) for the hand (green) and foot (yellow) and its relationship with the thalamopeduncular tumor of **Case B**. (**III**,**IV**): The brain MRI merged with the nTMS data shows that the cortical spinal tract (CST) runs antero-laterally with respect to a right thalamopeduncular tumor.

**Figure 2:**
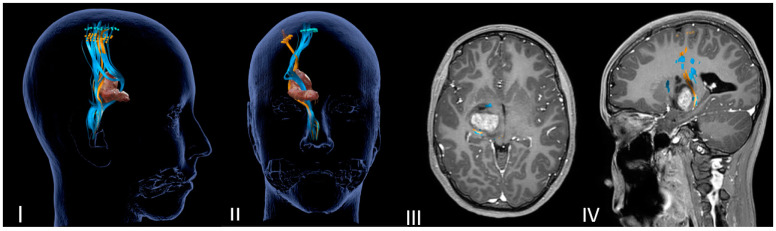
Neuronavigated transcranial magnetic stimulation pre-op study. (**I**,**II**) A 3D reconstruction of the nTMS data that shows cortical maps of the right spinal tract (CST) for the foot (blue) and hand (orange) and its relationship with the thalamopeduncular tumor of **Case A**. (**III**,**IV**) Brain MRI merged with nTMS data showing that the cortical spinal tract (CST) runs antero-medially respect to a right thalamopeduncular tumor.

DTI-MRI CST reconstruction combined with nTMS mapping allows for an accurate tridimensional visualization of the cortical spinal bundles and helps the surgeon to choose the safer surgical corridor (Figure 1 and Figure 2).

In addition to the location and size of the lesions, the DTI MRI and the nTMS data are important tools used to decide the surgical approach.

We performed three main surgical approaches: transsylvian, transcortical transtemporal, and transcortical parietal.

Furthermore, intraoperative neurophysiological monitoring (corticospinal and corticobulbar SSEPs and MEPs) and cortical-subcortical mapping [10] guided all the intraoperative steps of the surgery.

**Figure 3:**
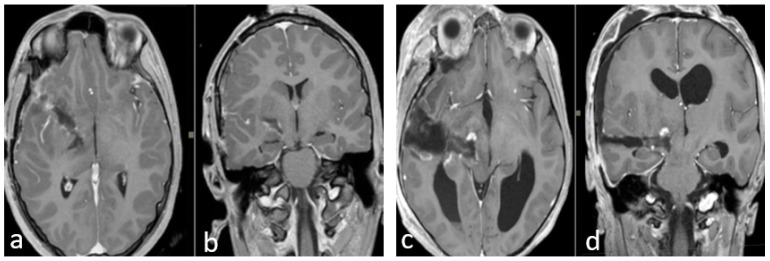
(**a**–**d**) Early postoperative brain MRI with gadolinium. (**a**,**b**) An early postoperative brain MRI, T1-weigthed with gadolinium in the axial (to the **left**) and coronal plane (to the **right**), for **Case A** in Figure 2. (**c**,**d**) An early post operative MRI, T1-weigthed with gadolinium, for **Case B** in Figure 1. **In detail**: In accordance with the nTMS data, we performed a transsylvian approach for **Case A** (**a**,**b**) obtaining a gross total resection (**GTR**). In **Case B** (**b**–**d**), we performed a trans-temporal approach, obtaining a subtotal resection (**STR**).

Finally, in all cases, we performed a postoperative brain MRI with gadolinium within 24 h of the surgery (Figure 3).

## 4. Results

In the last 17 years (from 2005 to 2022), a total of eight children with thalamopeduncular tumors underwent surgery in the Academic Pediatric Neurosurgery Department of Padova and in the Academic Neurosurgery Department of Verona.

In our series, the mean age was 9 years (range 3–15 years), with no gender prevalence, and the patients had not undergone any previous surgical intervention. Six patients developed “thalamo-peduncular syndrome” [11] with progressive spastic hemiparesis, and in three of them, facial cranial nerve palsy was observed. Other symptoms at onset were headache and an unstable gait. Indeed, three patients presented chronic hydrocephalus that did not require shunt surgery before the tumor resection.

In seven patients, the tumor was unilateral (in four on the left side and in three on the right side), and it was bilateral in one case. In all cases, the preoperative brain MRI with gadolinium showed mixed solid-cystic lesions with non-homogeneous contrast enhancement. 

Preoperative DTI-MRI showed a posterior displacement of the cortical spinal tracts in three cases (posterolateral in) and anterior (four antero-lateral and one antero-medial) displacement in five cases.

In the most recent cases (**Case A**–**Case B**), the preoperative nTMS was performed to obtain a cortical motor mapping and the cortical spinal bundles reconstruction. The nTMS data confirmed the DTI MRI data for the motor bundles’ displacement, adding functional information about cortical and subcortical motor areas of the mouth, arms and legs (Table 3).

A total of 11 procedures were performed on eight patients. Two patients (**Case E**–**Case H**) required a second surgery for progression one and four years after surgery, respectively, and one required an early second-look surgery due to a partial resection (**Case D**).

The main surgical corridor used was the transsylvian approach (**Case A**–**E**–**F**–**G**–**H**). In two cases, we used the transcortical transtemporal route (**Case B**–**C**), and in one case, we used a posterior parietal approach (**Case D**) due to the greater posterior parietal development of the lesion. We achieved GTR in two cases (**Case A**–**Case C**), STR in five (**Case B**–**E**–**F**–**G**–**H**), and PR in one (**Case D**).

All the tumors were pilocytic astrocytoma, but in one, we found a more infiltrative and aggressive histopathological pattern with a higher proliferation index (Ki67) (**Case D**) (Table 4).

The location and the extension of the tumor together with the DTI-MRI and nTMS reconstruction CST data guided the preoperative surgical plan. In all cases, we used intraoperative neurophysiological monitoring of the SSEPs, MEPs, and cortical\subcortical mapping. The brainstem acoustic evoked responses (BAERs) were used in the presence of a great extension of the lesion to the midbrain and the pons and the visual evoked potentials (flash-VEPs) in cases of the involvement of the optic chiasm or optic tracts.

Two patients showed improved neurological status after the surgery (**Case B**–**G**) and four maintained stable (**Case A**–**C**–**E**–**H**). Two patients worsened immediately after the surgery (**Case D**–**F**), but one (**Case F**) improved during recovery, recovering to his pre-surgery neurological status after a period of rehabilitation.

Three patients had a progression of their disease (**Case E**–**D**–**H**). Two patients (**Case E**–**H**) experienced a regrowth of the lesion after 15 months and four years, respectively, and therefore underwent an additional surgery, obtaining a new subtotal resection with good clinical and radiological long-term outcomes.

In only one case (**Case D**), due to an important extension and aggressive histopathological pattern, a partial resection was achieved. For this reason, we performed an early second-look surgery to obtain a new partial resection. Therefore, this patient received additional adjuvant therapy (CHT\RT) after a multidisciplinary discussion with the oncologist group. Finally, this patient died due to a rapid progression of disease. 

At the last neuroradiological and clinical follow-up, five patients had a stable residual tumor (**Case B**–**E**–**F**–**G**–**H**), two were disease-free (**Case A**–**C**), and one was deceased (**Case D**). The mean follow-up was 64.5 months (range 10–120 months).

## 5. Discussion

**Figure 4:**
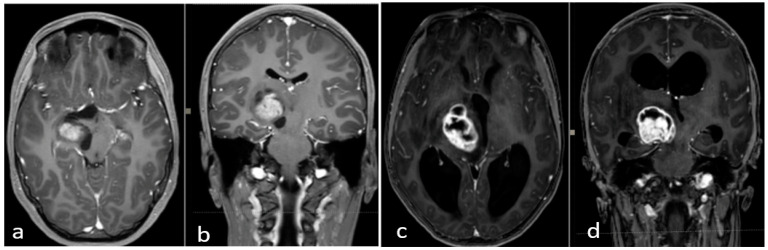
Preoperative brain MRI of the thalamopeduncular tumor of **Case A** (**a**,**b**) and of **Case B** (**c**,**d**). (**a**,**b**) Brain MRI, T1-weighted with gadolinium, showing solid-cystic right thalamopeduncular tumors in the axial plane (on the **left**) and coronal plane (on the **right**) in **Case A**. (**c**,**d**) Brain MRI, T1-weighted with gadolinium, showing a right thalamopeduncular lesion with disomogenous contrast enhancement in the axial (on the **left**) and coronal (on the **right**) plane in **Case B**.

Thalamopeduncular tumors are lesions that arise at the junction between the thalamus and the cerebral peduncles [1] (Figure 4). Most of these tumors are slow-growing pilocytic astrocytoma and displaced the corticospinal bundle, leading to the typical contralateral progressive spastic hemiparesis described as “childhood thalamopeduncular syndrome” [11].

The natural history of pediatric low-grade gliomas is favorable, and children have an excellent survival prognosis in comparison to adults. Overall survival rates at 10 and 20 years range from 80 to 90%. Therefore, in the pediatric population, these lesions rarely undergo malignant transformation during their lifetime and have a very low mortality rate [12,13]. For all these reasons, these patients can reach later adulthood.

Our series collected and retrospectively reviewed eight children with thalamopeduncular tumors who underwent surgery in the Academic Pediatric Neurosurgery Departments of Padova and Verona. In most cases, the clinical presentation observed was “childhood thalamopeduncular syndrome”, as described in the literature [4,5,6,7,8,9,10,11]. Other symptoms such as headache and cranial nerve palsy were observed. Two cases (**Case B**–**F**), due to persistent hydrocephalus, required ventricular-peritoneal shunt surgery after tumor resection, unlike in the Baroncini and Cinalli series [14,15].

All patients in our series underwent elective surgery. We achieved gross total resection in two patients, subtotal resection in five, and a partial resection in one case (<90% removal). No mortality related to surgery occurred.

All tumors were pilocytic astrocytoma and were examined for their proliferation activity using a Ki67 marker with a mean of 2% (range 1–5%).

Three patients underwent an additional surgery for tumor progression, obtaining a new subtotal resection in two and a partial resection in one. This latter case presented a more infiltrative histopathological pattern, with a Ki67 of 5%, and a more rapid progression of the disease. For this reason, we performed an early second-look surgery and then an additional chemotherapy regime with carboplatin and vincristine followed by radiotherapy (SIOP-LGG 2004 protocol) [16].

Indeed, one patient died due to rapid progression of their disease, while the other seven patients in our series had a stable residual tumor or were disease-free at the last follow-up.

Our surgical series provides further evidence regarding the key role of surgical treatment with curative intent for challenging entities such as thalamopeduncular tumors with a low-grade histology.

According to the literature [17,18], due to the very low mortality rate of this condition and the rare possibility of undergoing malignant transformation, in low-grade thalamopeduncular tumors, we suggest maximizing tumor resection as the treatment of choice. The cornerstone of treatment of these lesions is surgery, even in cases of tumor regrowth.

**Figure 5:**
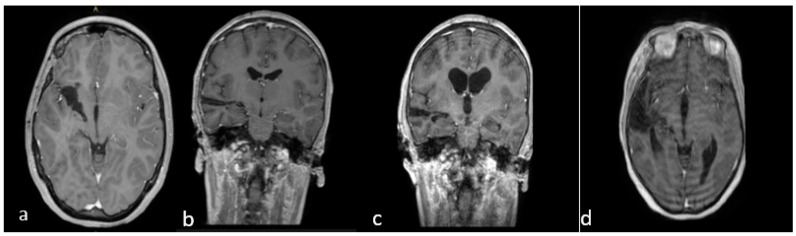
(**a**–**d**) Brain MRI, T1-weighted with gadolinium. Last follow-up of **Case A** (**a**,**b**) and **Case B** (**c**,**d**). (**a**,**b**) Brain MRI, T1-weighted with gadolinium, in the axial and coronal planes of **Case A**, showing good control of the disease (2021). (**b**–**d**): MRI, T1-weighted with gadolinium, in the coronal and axial planes of **Case B**, showing no evidence of relapse five years post-op.

Even a subtotal resection offers a good long-term outcome, as shown in our series. Five cases with subtotal resection were clinically and radiologically stable at long-term follow-up (Figure 5).

Preoperative advanced techniques of neuroimaging, including the more recent nTMS, are important tools used to plan surgery and guide the intraoperative steps of tumor resection with a good risk–benefit ratio. Therefore, the surgical corridor is chosen according to the tumor location and size and according to the relationship between the tumor and the corticospinal tract, studied with DTI MRI and nTMS [19].

The introduction of nTMS for pediatric thalamopeduncular tumors has led to additional information about the topography of motor pathways from the brain cortex and provides functional information about the different components inside the cortical spinal bundles. Thus, the integration between anatomic and functional studies based on DTI MRI and nTMS allows for more precise and detailed knowledge of the motor bundles’ position with respect to the tumor and consequently allows us to tailor a better surgical approach.

nTMS is also useful, as reported in the recent literature, for better understanding the benefits and risks of surgery in terms of neurological functional outcomes and to convey realistic expectations to families [7].

The use of an nTMS machine on children requires some precautions, such as the adaption of the nTMS seat workstation to the size of the children. The seat of the nTMS workstation is too large for a young patient, and so it must be adjusted to the height of the child in each individual case.

Therefore, nTMS is an exam that requires the patient’s concentration and collaboration, which is difficult in the case of young patients, but we did not encounter any problems during mapping, and no side effects were registered for our patients, as the latest systematic review of the literature describes [5].

Even though further studies are needed to verify the utility of nTMS so as to improve surgical and functional outcomes in pediatric thalamopeduncular tumors, this paper and another one recently published by our work team describe the first surgical series where nTMS was applied in pediatric thalamopeduncular tumors for a preoperative study of the arrangement of the corticospinal bundles [20].

The technological tools described are valid instruments used to preserve and, in some cases, improve neurological function in patients with thalamopeduncular low-grade gliomas.

The limitations of this study must be kept in mind due to the small number of patients presented. Furthermore, our paper does not have statistical and comparative value with respect to overall survival with and without the use of these tools, but we wish to stress the utility of advanced presurgical tools for reconstructing cortical spinal bundles, as nTMS and DTI MR allow us to perform surgery on lesions located in deep and complex areas of the brain.

Indeed, we underline our surgical attitude to planning a maximal safe resection using DTI MRI, as described above, in children harboring thalamic and thalamopeduncular low-grade gliomas, as well as the use of innovative techniques as nTMS.

According to our experience, many residual tumors can be observed with serial MRI, and upon progression or clinical manifestation, further surgery and eventual oncological therapy can be considered depending on the histopathological and molecular tumor features, the location of the tumor and the patient’s functional status.

The innovative technique described allows us to choose a safer surgical approach and helps the surgeon to obtain good control of the disease.

Another important aspect observed in children with thalamopeduncular tumors is that even though immediate worsening in the postoperative neurological exam is possible, in most patients, an improvement of their clinical status can occur during recovery or with a brief rehabilitation period.

As suggested in the literature [21], this clinical behavior in patients with thalamopeduncular tumors is probably due to a reduction in the compressive effects of the thalamopeduncular tumor on the thalamus and on the motor spinal tracts.

## 6. Conclusions

In the past, thalamopeduncular tumors were considered inoperable, and their surgery was characterized by elevated morbidity and mortality. Nowadays, safe surgical planning with advanced preoperative techniques, such as DTI MRI and nTMS, and intraoperative tools, such as intraoperative neurophysiological monitoring and neuronavigation systems, is the key to obtaining good control of the disease with acceptable surgery-related morbidity.

Therefore, due to the potential indolent behavior of thalamopeduncular low-grade gliomas, neurosurgical attitudes have changed, becoming more aggressive in recent years.

By using DTI MRI and nTMS for pediatric thalamopeduncular tumors, we showed a feasible and curative surgical approach which allowed us to navigate areas previously considered as a neurosurgical taboo, for it was impossible to conduct even a subtotal resection without severe complications, as supported by the recent literature.

Even if the use of nTMS is in its initial stages and further studies are needed to confirm its real potential for support in these kinds of tumors, our experience suggests the utility of nTMS as a preoperative instrument to provide functional information for cortical spinal bundles observed using DTI MRI, allowing us to improve the functional outcomes of patients with thalamopeduncular tumors.

Finally, we underline that a small residual tumor can be stable for many years, even after a subtotal resection; thus surgery for thalamopeduncular tumors should be encouraged.

## Figures and Tables

**Table 1:** Data series.

Case	Sex; Age	Histology	Pre-Op EON	HY	Treatment	Post-Op EON	Relapse\ Progression	2nd Treatment
**A**	F; 12	PA	HH	N	Surgery	HH Stable	N	-
**B**	F; 13	PA	Mild HP	Y	Surgery	HP improved	N	-
**C**	M; 7	PA	Moderate HP; VII CN palsy	N	Surgery	HP and VII CN palsy stable	N	-
**D**	F; 3	PA *	Ataxia	Y	Surgery	Worsened severe HP	Progression	Additional surgery + CH\RT
**E**	M; 15	PA	Mild HP	N	Surgery	HP stable	Progression	Additional surgery
**F**	F; 9	PA	Moderate HP	Y	Surgery	Transient HP Worsened **	N	-
**G**	M; 8	PA	Mild HP; VII CN palsy	N	Surgery	HP and VII CN palsy improved	N	-
**H**	M; 6	PA	HP	N	Surgery	HP stable	Progression	Additional surgery

**EON**, neurological exam; **HP**, hemiparesis; **HH**, headache; **PA**, pilocytic astrocytoma; PA *, more aggressive histopathological pattern (Ki67 > 5%); **CN**, cranial nerve; **HY**, hydrocephalus; **Y**, yes; **N**, no. **Transient Worsened** **, EON improved just during recovery and after a brief rehabilitation period.

**Table 2:** Surgical approaches performed, level of tumor resection and follow-up.

Case	Surgical Approach	EOR	FU (MO)	LTFU
**A**	Transsylvian	GTR	11	Disease free
**B**	Transtemporal T1–T2	STR	10	Stable residual
**C**	Transtemporal T1–T2	GTR	96	Disease free
**D**	Parietal transcortical	PR	36	Deceased
**E**	Transsylvian	STR	108	Stable residual
**F**	Transsylvian	STR	120	Stable residual
**G**	Transsylvian	STR	84	Stable residual
**H**	Transsylvian	STR	51	Stable residual

**EOR**, amount of tumor resection; **GTR**, gross total resection; **STR**, subtotal resection; **PR**, partial resection; **FU**, Follow-up; **MO**, months, **LTFU**, long-term follow-up.

**Table 3:** DTI and nTMS data for cortical spinal tracts’ position with respect to the thalamopeduncular tumor.

Case	DTI CST	nTMS CST
**A**	AM	AM And P
**B**	AL	AL And P
**C**	AL	N\A
**D**	AL	N\A
**E**	PL	N\A
**F**	P	N\A
**G**	P	N\A
**H**	AL	N\A

**DTI**, diffusor tensor imaging; **nTMS**, neuronavigated transcranial magnetic stimulation; **CST**, cortical spinal tract; **AL**, antero-laterally; **AM**, antero-medially; **P**, posterior; **N\A**, data not available.

**Table 4:** Histopathological report and molecular features.

Case	Histology Features	ki67%	kiaa1549-braf	braf v600e
**A**	PA (Nos)	2%	positive	negative
**B**	PA (Nos)	1%	positive	negative
**C**	PA (Nos)	1%	(-)	(-)
**D**	PA (infiltrative)	5%	negative	negative
**E**	PA (Nos)	3%	(-)	(-)
**F**	PA (Nos)	1%	(-)	(-)
**G**	PA (Nos)	1%	negative	negative
**H**	PA (Nos)	3%	(-)	(-)

**PA** pilocytic astrocytoma; **Nos** not otherwise specified; (-) Data non avaible.

## Data Availability

Not applicable.
